# A Survey of Scale Insects (Sternorryncha: Coccoidea) Occurring on Table Grapes in South Africa

**DOI:** 10.1673/031.009.4701

**Published:** 2009-06-26

**Authors:** Vaughn M. Walton, Kerstin Krüger, Davina L. Saccaggi, Ian M. Millar

**Affiliations:** ^1^ 4079 Ag and Life Sciences, Building Oregon State University, Corvallis, OR 97331-7304, U.S.A; ^2^ Department of Zoology & Entomology, University of Pretoria, Pretoria, 0002 South Africa; ^3^ ARC-Plant Protection Research Institute, Private Bag X134, Queenswood, Pretoria, 0121 South Africa

**Keywords:** mealybugs, soft scale insects, Pseudococcidae, distribution, quarantine, post harvest pests

## Abstract

Increasing international trade and tourism have led to an increase in the introduction of exotic pests that pose a considerable economic threat to the agro-ecosystems of importing countries. Scale insects (Sternorryncha: Coccoidea) may be contaminants of export consignments from the South African deciduous fruit industry to the European Union, Israel, United Kingdom and the United States, for example. Infestations of immature scale insects found on South African fruit destined for export have resulted in increasing rates of rejection of such consignments. To identify the risk posed by scale insect species listed as phytosanitary pests on table grapes to the abovementioned importing countries, a field survey was undertaken in 2004–2005 in vineyards throughout all grape-producing regions in South Africa. Coccoidea species found during the current field survey were *Planococcus ficus* (Signoret), *Pseudococcus longispinus* (Targioni Tozzetti), *Coccus hesperidum* L. and *Nipaecoccus viridis* (Newstead). With the exception of *Pl. ficus*, which has only been collected from *Vitis vinifera* (Vitaceae) and *Ficus carica* (Moraceae) in South Africa, these species are polyphagous and have a wide host range. None of the scale insect species found to occur in vineyards in South Africa pose a phytosanitary risk to countries where fruit are exported except for *Ferrisia malvastra* (McDaniel) and *N. viridis* that have not been recorded in the USA. All scale insects previously found in vineyards in South Africa are listed and their phytosanitary status discussed. The results of the survey show that the risk of exporting scale insect pests of phytosanitary importance on table grapes from South Africa is limited.

## Introduction

The South African table grape export industry is situated in mild Mediterranean and arid subtropical climates. Several scale insect (Sternorryncha: Coccoidea) species have successfully colonized hosts, including grapevine (*Vitis vinifiera* L. (Vitaceae)), in these climates (Ben-Dov 1994). More than 80% of table grape production in South Africa occurs in the Western Cape region. Other production areas include the Northern Cape, Eastern Cape, Limpopo, Free State and Mpumalanga. During 2003, South African table grape exports totaled 198,264 metric tons (Hugh Campbell, Deciduous Fruit Producers Trust (DFPT) research, personal communication). Most table grapes were exported to central Europe (40%), followed by Great Britain (35%), Asia and the Far East (11%), the USA (4%), the Middle East and Mediterranean countries (6%) and other African countries (4%) (Hugh Campbell, personal communication).

Many mealybug (Pseudococcidae) species, such as *Planococcus ficus* (Signoret), the key pest on grapevines in South Africa, are able to complete their entire lifecycle on grapes (Walton and Pringle 2004). The almost year-long warm climate in most grape-growing regions in South Africa leads to overlapping generations of *Pl. ficus*, resulting in multiple life stages being present at any one time. Adult female scales on export grapes can be readily identified using morphological characters and consignments are often released following species identification. However, many consignments are rejected prior to shipment due to infestation with immature scale insects that are difficult to identify using morphological characters. As a result, immature scale insect identification tools have been developed in export-based fruit producing countries. These include both molecular (e.g. Beuning et al. 1999; Demontis et al. 2007; Saccaggi et al. 2008) and morphological (e.g. Gullan 2000; Wakgari and Giliomee 2005) methods. The risk of a quarantine pest occurring in certain production areas can be quantified by undertaking extensive area-wide field surveys. This study reports the results of an industry-wide field survey of scale insects in South African vineyards. In addition, a literature survey of scale insects previously reported on grapevines is presented.

## Meterials and Methods

To determine which scale insect species occur on table grapes, field surveys were conducted in different vine-growing regions in South Africa. Field surveys were undertaken from 1 November 2004 to 30 April 2005 as this is the period with the highest prevalence of scale insects in several grape-growing areas in the country (Whitehead 1957; Walton 2003). A portion (10%) of all producers in each grape-growing area in South Africa was randomly selected, and 10% of each production unit (farms with average sizes of between 30 and 100 hectares) was surveyed. A central systematic sampling system was used in vineyard blocks that ranged between three and ten hectares in size. One sample was collected in each block as follows: field scouts thoroughly inspected leaves, bunches, areas beneath the bark and areas in the root region in each of twenty evenly spaced plots consisting of five vines each. Whenever possible, scouts collected adult females in order to facilitate the identification process. Samples of between three and 50 scale insects from each block were placed into single vials containing 97% ethanol. Each vial was marked with GPS coordinates, production unit name, contact details, vine cultivar and sampling date.

Samples collected during the survey (2004–2005) were identified using molecular and morphological techniques. V. Walton carried out morphological identification of specimens using the Systematic Entomology Laboratory ARS, USDA Scale Insects Identification Tools for Species of Quarantine Significance (Miller et al. 2007). To confirm morphological identifications, mealybugs were also identified using a multiplex polymerase chain reaction (PCR) technique (Saccaggi 2006; Saccaggi et al. 2008) developed to identify the mealybugs *Pl. ficus, Planococcus citri* (Risso) and *Pseudococcus longispinus* (Targioni Tozzetti). *Pl. ficus* and *Ps. longispinus* are the most common mealybug species occurring in South African vineyards. *Pl. citri* has been historically associated with *V. vinifiera* in South Africa and is common in citrus groves in this country. Mealybugs that could not be identified morphologically, or whose identification was doubtful, were identified by I.M. Millar.

A literature search of scale insect species occurring on grapevines, was performed on the ScaleNet website, www.sel.barc.usda.gov/SCALENET/scalenet.htm, and used to determine historical records of these insects on vines in South Africa. (Ben-Dov et al. 2006). In addition, a query of ScaleNet was done to determine all scale insects found on *V. vinifiera* worldwide, followed by a crosssearch in order to determine which of these had been recorded in South Africa.

## Results and Discussion

Scouts collected 249 samples from all major grape-growing regions and 29 municipal districts during the survey (Table 1). Two hundred and twenty nine samples were identified using the morphological method by V. Walton and sub-samples were confirmed using the molecular identification method (128 samples). Mealybugs and scale insects that could not be identified morphologically by V. Walton were submitted for identification to I. M. Millar (20 samples). Of these, eighteen samples were identified as *Pl. ficus* and the remaining two were *Nipaecoccus viridis* (Newstead), collected in the Grootdrink municipal district (28.40 S 21.43 E) on ‘Thompson Seedless’ grapes, and *Coccus hesperidum* Linnaeus, collected in Groblershoop municipal district (28.53 S 21.59 E) on ‘Thompson Seedless’ grapes. Both species had not been previously recorded on *V. vinifiera*, and these are the first known records of these species from these areas.

**Table 1.**
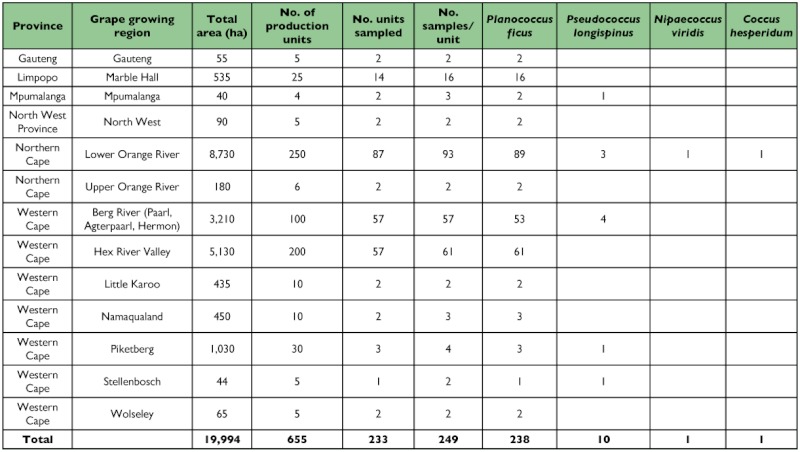
Grape-growing regions sampled for Coccidae in six Provinces in South Africa, their total area, approximate number of production units, number of units sampled, total number of samples collected and number of coccids found. Samples were collected between November and April.

The majority of species (95%) were *Pl. ficus*, a cosmopolitan mealybug reported from Europe, Israel and the USA among other regions. This species was found in the grape growing areas in South Africa (Table 1). Of the remaining samples, ten were *Ps. longispinus* (4%), one *N. viridis* and one *C. hesperidum* (1%). *Ps. longispinus* was found at six locations in the Western Cape Province: Paarl area (33.42 S 19.10 E, Berg River, 2 samples), Agterpaarl (33.40 S 18.54 E, Berg River, 1 sample), Hermon (33.24 S 18.58 E, Berg River, 1 sample) Stellenbosch (33.54 S, 18.50 E, 1 sample), and Piketberg (32.56 S 18.40 E, 1 sample). Further records are from the Northern Cape: Augrabies (28.40 S 20.26 E, Lower Orange River, 1 sample), Groblershoop (28.53 S 21.59 E, Lower Orange River, 1 sample) and Kanoneiland (33.24 S 18.58 E, Lower Orange River, 1 sample) and Mpumalanga: Groblersdal (25.10 S 29.25 E, 1 sample). These are the first records from these localities.

The online literature search listed 1389 records of scale insect species found in South Africa (Ben Dov et al. 2006), including 281 species from the Pseudococcidae. A total of 101 scale species have been recorded on *V. vinifiera* worldwide (Ben-Dov et al. 2006). From this list, we have listed forty-six species recorded from a variety of host plants in South Africa (Table 2) as these species have the highest risk of occurring on grapevines in South Africa. Of these, 11 were recorded on *V. vinifiera* in South Africa. The following seven species belonging to the families Coccidae and Pseudococcidae were recorded during the survey: *C. hespendum, Cryptinglisia lounsburyi* Cockerell, *Trijuba oculata* (Brain), *Ferrisia malvastra* (McDaniel), *N. viridis, Pl. ficus* and *Ps. longispinus*.

In addition, four giant scale species (Margarodidae), all phytosanitary pests, have been recorded on *V. vinifiera* in South Africa (Table 2). *Margarodes capensis* Giard, *Margarodes greeni* Brain, *Margarodes prieskaensis* (Jakubski) and *Margarodes vredendalensis* De Klerk, were studied and described by De Klerk et al. (1983). These species are subterranean and therefore not likely to be found on fruit destined for export. Both *T. oculata* and *C. lounsburyi* are of phytosanitary concern but are not found in any country to which South Africa exports table grapes (Ben-Dov et al. 2006). Whereas *F. malvastra* and *N. viridis* are found in all countries importing fruit from South Africa except the USA, both species have been reported from Mexico (Ben-Dov et al. 2006). Only single samples of *N. viridis* and *C. hesperidum* were collected in the survey, suggesting that they are incidental species in vineyards in South Africa. *C. hesperidum* is cosmopolitan and occurs in Australia, China, Egypt, Europe, Guam, Hawaii, India, Iran, Iraq, Israel, Jordan, Mexico, Pakistan, South Africa, and the USA (Ben-Dov 1994; Longo et al. 1995).

**Table 2.**
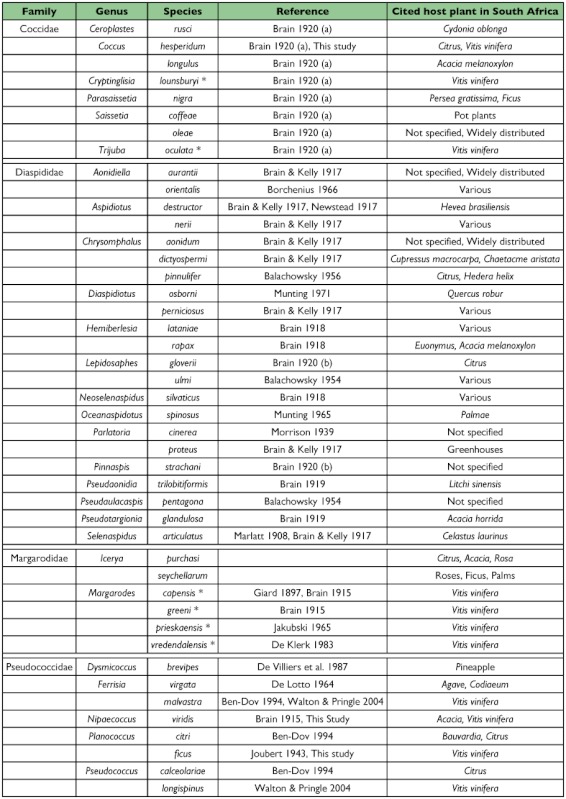
Records of scale insects found on *Vitis vinifera* worldwide, and thqt occur on grapevines and/or other hosts in South Africa. Currently, eleven species are found on *V. vinifera* in South Africa, and six of these (indicated with an asterisk) are of phytosanitory concern to other parts of the world. Source: Ben Dov et al. 2006.

**cont.**
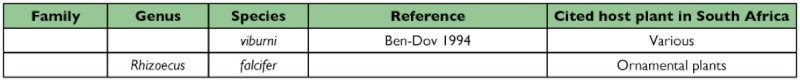

This survey confirms results of previous studies which found that *Pl. ficus* is dominant in South African vineyards (Kriegler 1954; De Lotto 1975), whereas *Ps. longispinus* is less abundant. These two cosmopolitan species are now resident in all countries importing grapes from South Africa and, therefore, do not pose a phytosanitary risk (Ben-Dov 1994; Walton and Pringle 2004). However, *F. malvastra* and *N. viridis* were during the survey and have not been recorded in the USA. Thus, with this exception, we conclude that the risk of exporting exotic scale insect pests to novel geographic regions on table grapes is limited. We also conclude that the multiplex PCR method is an ideal tool for accurately identifying common mealybug species such as *Pl. ficus* so that bulk shipments of table grapes containing these species can be cleared for export, although samples containing other species will need to be identified using morphological techniques.
